# Effect of silicone oil versus gas tamponade on macular layer microstructure after pars plana vitrectomy for macula on rhegmatogenous retinal detachment

**DOI:** 10.1186/s12886-024-03377-x

**Published:** 2024-03-14

**Authors:** Abdulaziz Mohammed Al-Shehri, Saud Aljohani, Khalid Abdulaziz Aldihan, Musa Johaiman Alrashedi, Saad Alrasheed, Patrik Schatz

**Affiliations:** 1 Vitreoretinal Division, King Khaled Eye Specialist Hospital, Riyadh, Saudi Arabia; 2 Surgery Department, Taif university, Taif, Saudi Arabia; 3 Ophthalmology Department, Imam Abdulrahman Bin Faisal University, Dammam, Saudi Arabia; 4 Fellowship and Residency Training Program, King Khaled Eye Specialist Hospital, Riyadh, Saudi Arabia; 5 Diagnosic imaging department, King Khaled Eye Specialist Hospital, Riyadh, Saudi Arabia; 6 Department of Ophthalmology, Clinical Sciences, Skane University Hospital, Lund University, Lund, Sweden

**Keywords:** Macular thickness, Sub foveal choroidal thickness, Silicone oil tamponade, Gas tamponade, Rhegmatogenous

## Abstract

**Purpose:**

To analyze structural changes in the macular retinal layers and sub-foveal choroidal thickness (SFCT) in eyes after macula-on rhegmatogenous retinal detachment (RRD) repair by pars plana vitrectomy with either silicone oil (SO) or gas tamponade, and the effect of these changes on visual acuity.

**Patients and methods:**

Retrospective study which included 26 eyes in the SO Group and 32 in the Gas Group. Optical coherence tomography (OCT) scans of the affected eyes were obtained before surgery, and 3 months after PPV in the Gas Group, and during silicone oil in situ and 3 months after SO removal, in the SO Group. Qualitative assessment of photoreceptor layer and foveal contour, along with quantitative assessment of macular retinal thickness and SFCT was performed. Postoperative OCT macular microstructural changes were recorded and correlated to corrected distance visual acuity (CDVA). Intraocular pressure (IOP) was measured preoperative and at 3 months post operative.

**Results:**

There was a 2-line loss (from 20/28 preoperatively to 20/40 at final follow-up) of CDVA in the SO Group (*p*=0.051), while there was no statistically significant change in CDVA in the Gas Group (*p*=0.786). There was no significant correlation between CDVA loss and duration of silicon tamponade (*r*=-0.031, *p*=0.893). There was a statistically significant increase in IOP from its baseline to final follow-up of 0.7 mmHg in the SO Group (*p*=0.023) while there was no statistically significant change in IOP in the Gas Group. During silicone oil tamponade, there was approximately 11% and 5% of retinal and sub-foveal choroidal thinning respectively, which was moderately resolved following silicone oil removal. 20% (5/24) of eyes in the SO Group had qualitative flattening of foveal contour during SO tamponade that resolved after SO removal.

**Conclusion:**

Thinning of the macula was noticed after macula-on RRD repair with SO tamponade. Such thinning was only partially reversible after the removal of SO.

## Introduction

Rhegmatogenous retinal detachments (RRDs) are often repaired using gas and silicone oil (SO) as intraocular tamponades [1]. Multiple studies over the years suggest that gas and SO to be effective and safe for this purpose [2, 3]. However, a potential toxicity of SO to the human retina has been explored and remains controversial [4, 5]. There are several potential causes of vision loss following RRD repair using a gas or SO endo-tamponade, including changes of intraocular pressure (IOP) (intra- and post-operatively), cystoid macular oedema, epiretinal membrane formation and recurrent RRD. Retinal toxicity is also a potential cause of loss of vision [1]. There are also reports of visual loss with unknown etiologies occurring after the SO tamponade has been removed [6–8]. The loss of vision in these cases has been attributed to thinning of the inner retinal layers in some studies [1, 9, 10], this conclusion is contested [11]. The validity of the conclusions of many of the case studies exploring this phenomenon is limited by being small-scale studies with few participants, and lack of control cohorts, using only the individual’s untreated eye as a control [1]. Yet this approach might be flawed, because the volume and thickness of the macula can vary appreciably between the two eyes of the same healthy individual [11, 12].

The aim of this study was to investigate the effect of gas and SO tamponades upon the microstructure of the macula following RRD repair using pars plana vitrectomy (PPV). A secondary aim was to explore potential impacts of PPV and endo-tamponade upon the sub-foveal choroidal thickness (SFCT). In this study we analysed changes in retinal thickness and visual acuity over time of the same operated eyes as controls, comparing data obtained before surgery, during SO in situ, and after SO removal, and matched it to corresponding data in eyes which received gas tamponade.

### Patients and methods

This is retrospective study has been conducted in accordance with the principles of the Declaration of Helsinki. Records (*N*=2,777) were retrieved for all patients who underwent PPV to treat RRD between 2015 and 2020. The records were evaluated individually to ensure they met specific inclusion and exclusion criteria. The inclusion criterion was that macula on RRD had been repaired using a gas or SO tamponade by PPV. Eyes that were excluded were ones that had combined tractional-RRD or recurrent RRD, grade C PVR or above, had been repaired by combining scleral buckle and PPV techniques, Optical coherence tomography (OCT) quality was low, had not been followed up or the pre- or post-operative data was incomplete. Patients with additional pathologies, such as glaucoma, diabetic macular oedema, epiretinal membrane, high myopia (> -6 diopters or > 26.1 mm axial length), macular scar, post-acute retinal necrosis, retinal dystrophy and treated endophthalmitis were also excluded from the study. Eyes that received heavy silicone oil as tamponade, underwent scleral buckling surgery or pneumatic retinopexy were not included in this study.

Once inclusion and exclusion criteria had been applied, a total of 58 eyes were included in the study. Of these, 32 eyes were from 32 patients who had undergone RRD repair using perfluoropropane (14 % C_3_F_8_) or sulfur hexafluoride (20% SF6) gas endotamponade; the remaining 26 eyes were from 26 patients, in whom the repair was carried out using SO.

The following patient data were recorded: age, diagnosis, gender, and surgery date. Other data that were extracted were the patients’ corrected distance visual acuity (CDVA) as determined by Snellen charts and intraocular pressure using Goldman applanation tonometry at two time points (pre-operative and at 3 months post tamponade removal). Also, this study considered the duration of SO tamponade, the findings of slit-lamp examinations and whether the patient had a phakic lens or a pseudophakic lens.

Spectral domain optical coherence tomography (SD-OCT) images (Heidelberg Engineering, Inc, Heidelberg, Germany) were obtained of the central 6 mm of the macula of each eye. The OCT scans were conducted at three different time points for each patient in the SO group – prior to surgery, with the tamponade in place, and ≥3 months after the tamponade had been removed. For the patients in the gas tamponade group, OCT was performed before the procedure and 3 months after it. The Spectralis OCT device automatically measures the thickness of the retina in superior, inferior, nasal, and temporal macula, the central macula (CMT) and the macular volumetric. These data were recorded along with the total inner and outer retinal thicknesses. The total inner retinal thickness was determined from the central 1 mm of the macula, taken as the distance from the outer plexiform layer to the retinal nerve fiber layer. Meanwhile, the total retinal thickness was calculated as being the distance from the retinal pigment epithelial cell (RPE) to the internal limiting membrane, in the central 1 mm of the macula.

SD-OCT was used to obtain SFCT measurements prior to surgery, during SO and 3 months following the removal of the SO tamponade.

The OCT software’s manual caliper facility was used to measure the choroidal thickness. The choroidal thickness was defined as the distance between the hyperreflective line corresponding to the base of retinal pigment epithelium and the margin or hyperreflective line corresponding to choroidal-scleral interface. Two independent retina specialists collected all the measurements; in this work, we have used the mean values of the specialists’ measurements.

### Surgical procedures

Surgeries were done by several different vitreoretinal surgeons. All patients had surgery within 24 hours of presentation. A standard 3-port, 23-gauge PPV with endolaser photocoagulation and SO or gas tamponade were performed. The indications for using an SO rather than a gas tamponade was the presence of multiple or a very large retinal tears or air travel. When deemed necessary for optimal visualization during PPV, concomitant phacoemulsification and intraocular lens implantation were also performed. Subsequent surgery for removal of the SO along with phacoemulsification (if not done before) was timed according to the discretion of the treating physician and convenience of the patient. Removal of SO performed using 3-port, 23-gauge pars plana active SO removal. In general, the aim was to remove SO after 3-6 months if the retina attached and there was no traction on the retina.

### Statistical analysis

Corrected distance visual acuity (CDVA) was converted to logarithm of minimum angle of resolution (Log MAR) for the purpose of analysis. Statistical analysis was performed with IBM SPSS for windows (v.22; IBM Corp, Armonk, NY, USA). All figures were constructed with GraphPad Prism (version 8.4.3 for Windows, GraphPad Software, San Diego, California USA, www.graphpad.com.) The normality of the data was assessed by the Shapiro-Wilk test. Normally distributed preoperative and postoperative data was compared with paired t-test. Non-normally distributed preoperative and postoperative data was compared with Wilcoxon signed ranks test. Pre-silicon oil, silicon oil in situ, post silicon oil removal macular thickness in SO group was compared and preoperative and post operative macular thickness in gas group was also compared with one-way repeated measures analysis of variance (ANOVA). A p-value less than 0.05 was considered statistically significant.

## Results

Data from 58 eyes from 58 patients were collected. There were 32 eyes in the gas tamponade group and 26 eyes in the SO group.

### Patients demographics and clinical characteristics

Table 1 presents the demographic and clinical data for the two different tamponade treatment groups. The groups were similar in their distribution of the sexes and the median age of the participants. The majority of the patients (*n*=40) had no pre-existing medical conditions. There was no statistically significant difference in central retinal thickness at baseline between the SO 288±62 µm and Gas groups 277±25 µm (*p*=0.965). Also, there was no statistically significant difference in axial length between the SO 25.56±1.26 mm and Gas groups 24.35±1.16 mm (*p*=0.639). For SO group, the range in duration of the tamponade being in situ was 4−18 months (mean = 8.1±3.4 months). The average post operative follow-up duration for the gas group was 6-26 months (mean= 9.3±7.0 months), whilst for the SO group the average post operative follow-up duration was 5-40 months (mean = 11.6±11.0 months). Fourteen eyes (53.8%) of SO group had a phakic lens, whilst in the gas group, 23 eyes (71.9%) were phakic.

**Table 1:** Demographic and clinical characteristics

**Parameter**		**Silicone Oil Group**	**Gas (SF6 or C3F8) Group**
Patients %(n)		44.8% (26)	55.1% (32)
Age (years)		48.5±11.2 (23.0 to 69.0)	48.6±13.9 (21.0 to 71.0)
Gender % (n)			
	Male	66.7% (18)	65.6% (21)
	Female	33.3% (8)	34.4% (11)
Systemic disease % (n)			
	HTN DM Dyslipidemia Depression	11.5% (3) 7.6 %(2) 11.5% (3) 1.7 %(1)	12.5% (4) 12.5% (4) 3.1% (1) 0% (0)
RD quadrant % (n)			
	Superior	0% (0)	3.1% (1)
	Nasal	7.7% (2)	3.1% (1)
	inferior	19.2% (5)	0% (0)
	Temporal	3.8% (1)	21.9% (7)
	Superonasal	11.5% (3)	9.4% (3)
	Inferonasal	3.8% (1)	3.1% (1)
	Inferotemporal	34.6% (9)	12.5% (4)
	Superotemporal	19.2% (5)	46.9% (15)
Preoperative lens status			
	Phakic	53.8% (14)	71.9% (23)
	Pseudophakic	42.3% (11)	25.0% (8)
	Aphakic	3.8% (1)	3.1% (1)
Postoperative lens status			
	Phakic	3.8% (1)	18.8% (6)
	Pseudophakic	92.3% (24)	78.1% (25)
	Aphakic	3.8% (1)	3.1% (1)
Postoperative follow up (months)			
	Mean±SD	9.3±7.0	11.6±11.0
	Median (Range)	6.5 (6.0 to 26.0)	7.5 (5.0 to 40.0)
Preoperative Central macular thickness (µm)	Mean±SD	288±62	277±25
Tamponade indication			
	Inferior break 46.1% (12)		Superior temporal break 46.9% (15)
	Multiple break 38.4% (10)		Superior nasal break 34.3% (11)
	Giant retinal break 7.7% (2)		Temporal break 12.5% (4)
	Air travel 7.7% (2)		Nasal break 6.2.%. (2)
Silicone oil tamponade duration (months)			
	Mean±SD	8.1±3.4	
	Median (Range)	7.5 (4.0 to 18.0)	

*HTN* hypertension, *DM* diabetes mellitus, *RD* retinal detachment, *SF6 *Sulfur hexafluoride, *C3F8* Octafluoropropane

By the final postoperative follow-up, the majority of eyes in the SO group (92.3%) were pseudophakic, whilst only 78.1% were pseudophakic in the gas group. Whilst undergoing RRD repair one eye in the SO group also received cataract surgery and 13 eyes underwent concurrent phacoemulsification and intraocular lens implantation during SO removal. In contrast, in the gas group, three eyes underwent cataract surgery in conjunction with the RRD repair, whilst the other eyes in this group received cataract surgery after the RRD had been repaired, during a separate surgery. By the final follow-up, six eyes in the gas group (18.8%) and one eye in the SO group (3.8%) were still phakic.

### Macular retinal and choroidal thickness

**Fig. 1:**
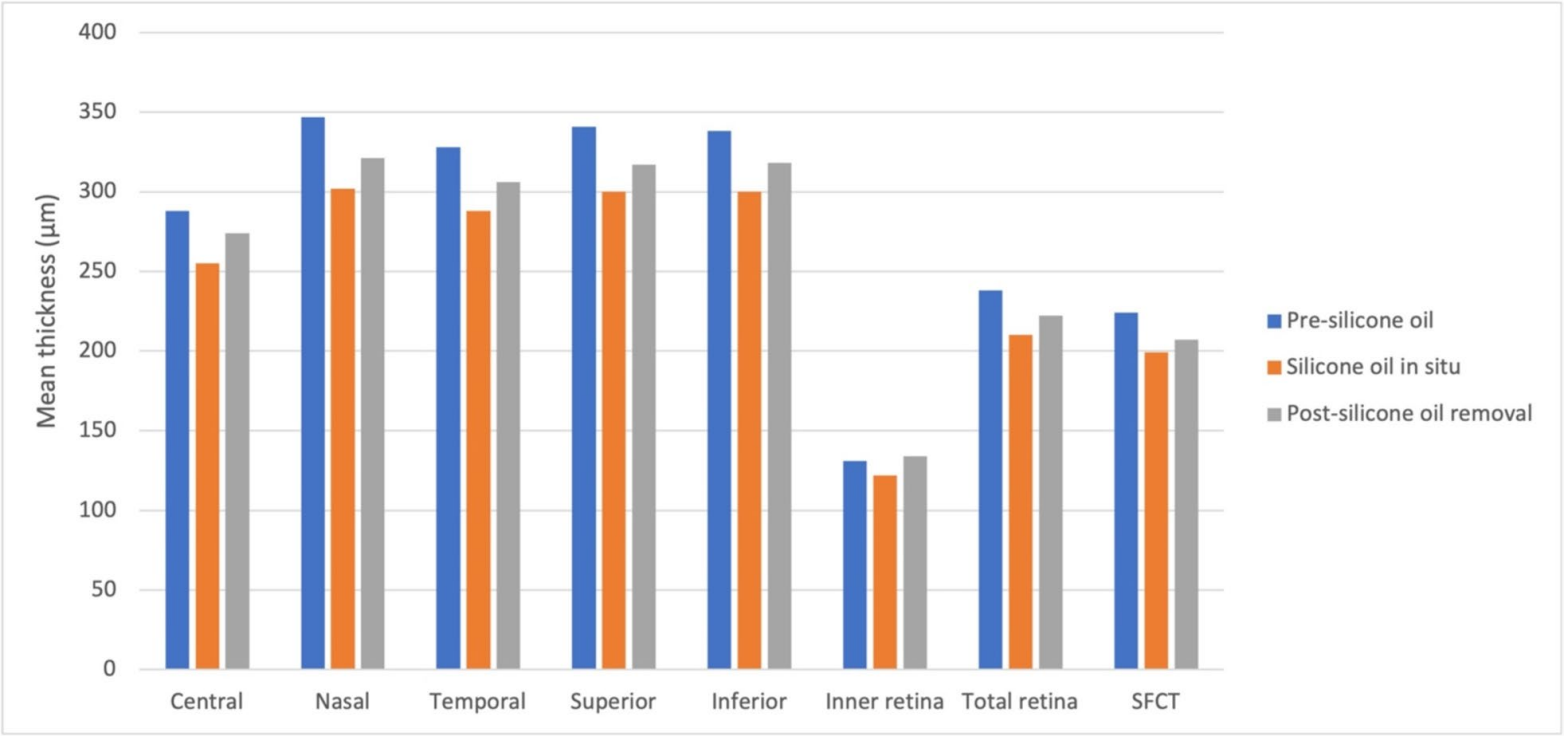
Macular retinal and choroidal thickness pre-silicone oil, silicone oil in situ, and post-silicone oil removal

Table 2 and Fig. 1 compare the thickness of the choroid and macula before applying SO, whilst the SO tamponade is in place and after its removal. Whilst the tamponade was in place, there was thinning of the total retinal and sub-foveal choroid (*p*<0.001); following removal of the SO, this was moderately resolved. The thickness of the inner retina did not change significantly. Moreover, we did further analysis of retinal thickness in these two eyes showing severe vision loss in SO group, which showed decreased retinal thickness during SO tamponade (250, 223, 235 µm in one eye and 290,245, 266 µm in the other eye, from before surgery to during SO tamponade and post SO removal, respectively).

**Table 2:** Macular retinal and choroidal thickness pre-silicone oil, during silicone oil, and post-silicone oil removal

**Parameter**		**Pre-silicone oil**	**Silicone oil in situ**	**Post-silicone oil removal**	*P*-value*
Retinal thickness					
	Central	288±62	259±29	275±27	0.066
	Nasal	347±34	305±26	320±26	<0.001
	Temporal	328±37	291±30	305±24	<0.001
	Superior	341±23	303±28	317±22	<0.001
	Inferior	338±35	301±28	316±28	<0.001
	Inner retina	131±15	126±37	136±32	0.335
	Total retina	238±74	214±40	224±32	0.217
Retinal volume		7.8±1.1	6.3±1.1	7.8±1.1	<0.001
SFCT		214±72	196±74	201±62	0.104

*SFCT* sub-foveal choroidal thickness

^*^one-way repeated measures analysis of variance (ANOVA). Retinal and choroidal thickness is given in µm and retinal volume in mm^3^

**Fig. 2:**
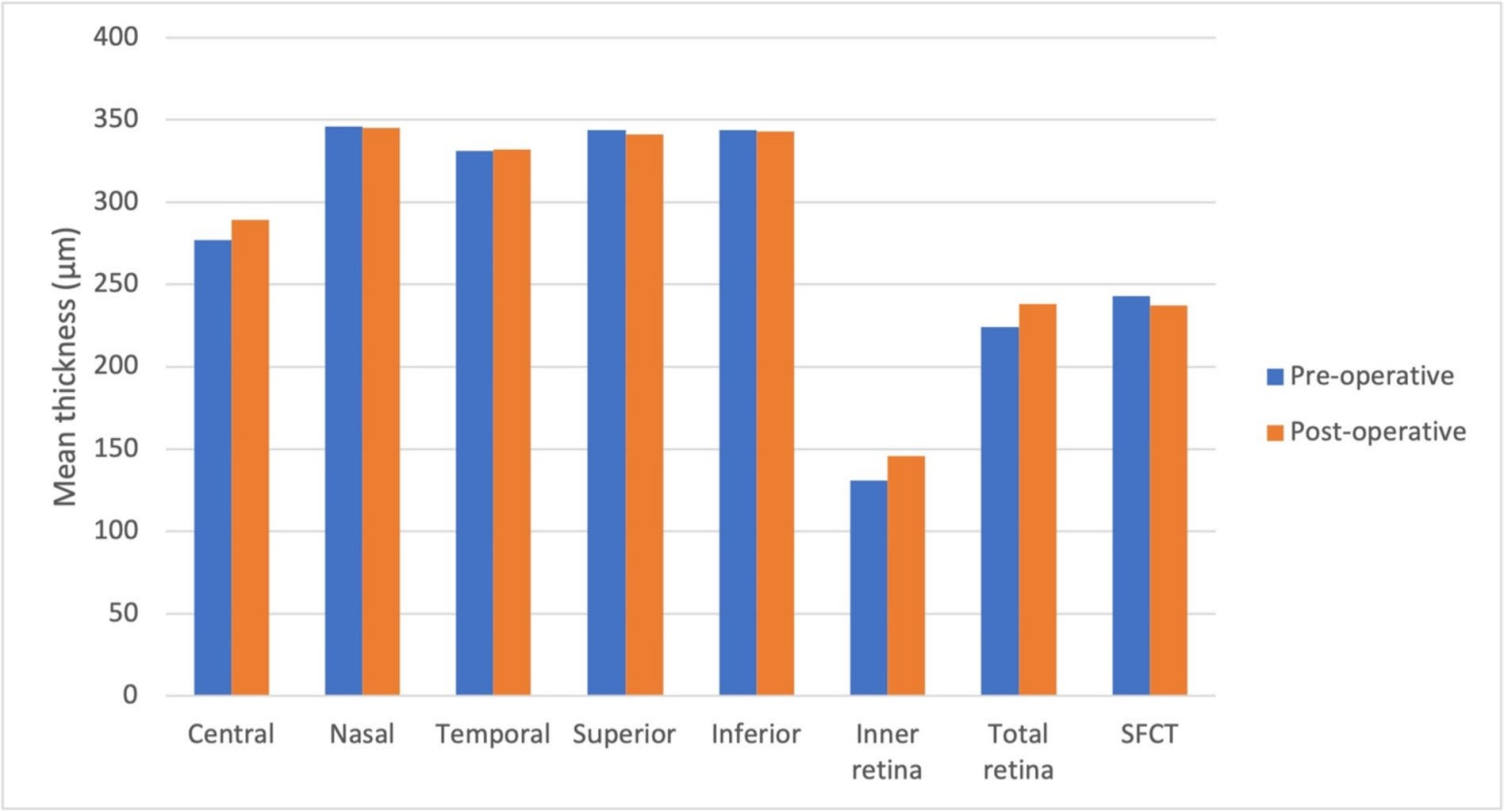
Macular retinal and choroidal thickness preoperative and postoperative in gas group

Similarly, Table 3 and Fig. 2 compare the choroidal and macular retinal thickness before and after the intervention in the gas group. After surgery, the central macula thickened by 12 µm (*p*=0.001), the inner retina thickened by ≥15 µm (*p*=0.002) and the total retina thickness increased by ≥14 µm (*p*=0.002). A further analysis of this group’s data did not expose any evidence of cystoid macular oedema or epiretinal membrane.

**Table 3:** Macular retinal and choroidal thickness pre and post-operative in Gas Group

**Parameter**		**Preoperative**	**Postoperative**	*P***-value**
Retinal thickness				
	Central	277±25	289±28	0.001**
	Nasal	346±20	345±23	0.537**
	Temporal	331±17	332±16	0.114**
	Superior	344±17	341±20	0.961**
	Inferior	344±25	343±21	0.828*
	Inner retina	131±22	146±30	0.002*
	Total retina	224±22	238±30	0.002*
Retinal volume		8.7±1.1	8.3±1.0	0.270**
SFCT		243±35	237±48	0.243**

*SFCT* sub-foveal choroidal thickness

^*^Paired *T*-Test

^**^Wilcoxon Signed Ranks Test. Retinal and choroidal thickness is given in µm and retinal volume in mm3

The statistical difference in the change in retinal volume was greater in the SO group (*p*=<0.001) than in the gas group (*p*=0.270).

**Fig. 3:**
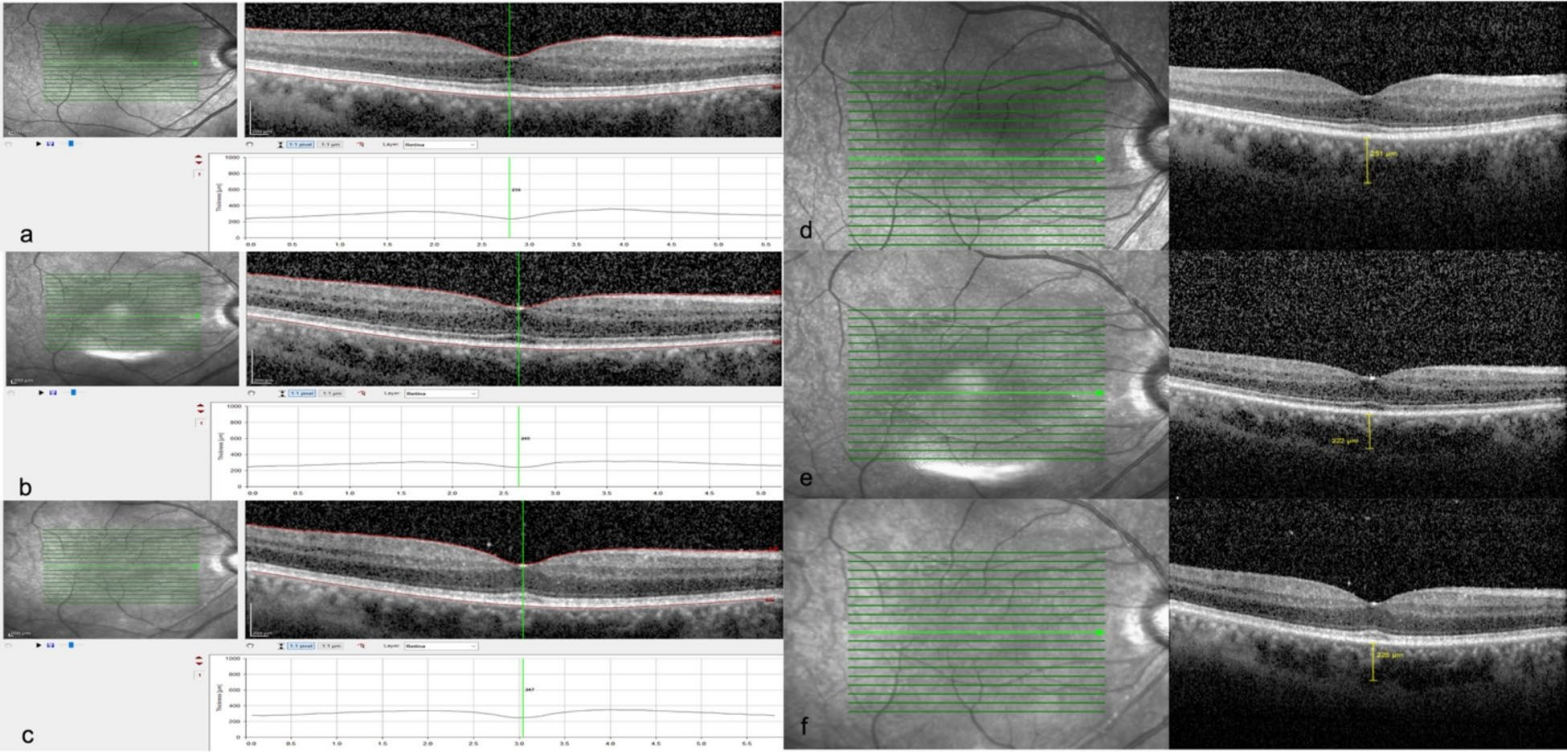
**a** Pre-operative optical coherence tomography scan of the macula of an eye with macula-on rhegmatogenous retinal detachment (top left panel). **b** The central macular thickness showed mild thinning during silicone oil tamponade (middle left panel). **c** central macular thickness returns to normal post silicone oil removal (lower left panel). **d** Corresponding sub foveal choroidal thickness measurements pre-operative (top right panel). **e** Sub foveal choroidal thickness during silicone oil tamponade (middle right panel). **F** Sub foveal choroidal thickness post silicone oil removal (lower right panel)

Figure 3 shows that as well as the quantitative and qualitative changes detected by OCT. Moreover, pre and post operative OCT of both groups showed 100 % qualitative intact foveal photoreceptor layer in all eyes. However, during SO tamponade, 20% (5/24) of the eyes in the SO group had a qualitative flattening of the foveal contour; however, once the tamponade was removed, the flattening was resolved.

### Visual acuity and intraocular pressure

**Fig. 4:**
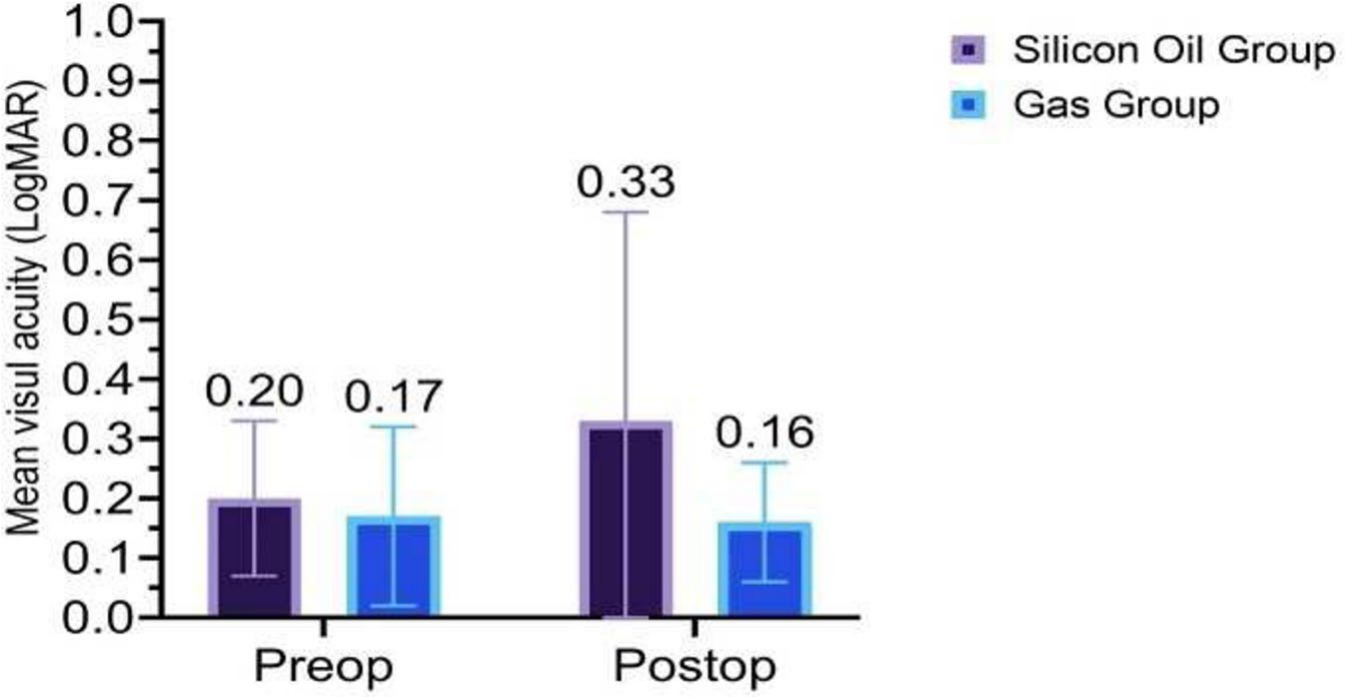
Corrected Distance Visual acuity Pre-operative and at final follow up

**Fig. 5:**
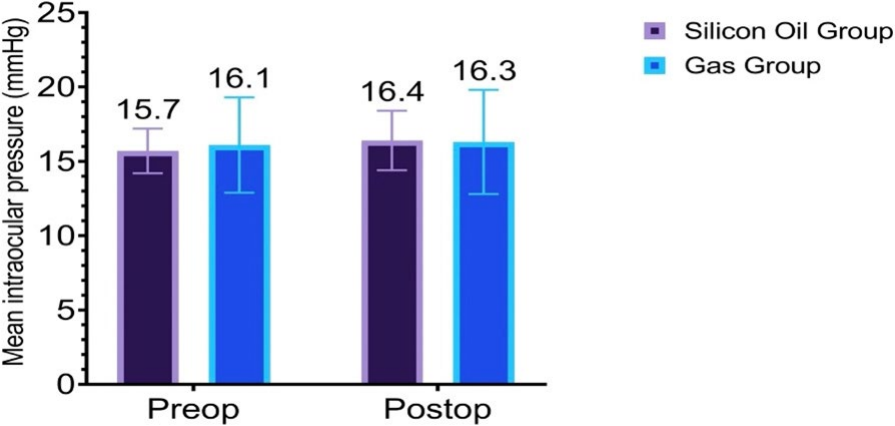
Intraocular pressure pre-operative and at final follow up

A comparison of the pre- and post-operative CDVA and IOP data for the SO and gas groups is presented in Table 4. It shows that in the SO group, there was a postoperative 2-line loss (from 20/28 to 20/50) of CDVA (*p*=0.051) with large variability (SD=0.037) (Fig. 4). There was no significant correlation between CDVA loss and duration of silicon oil tamponade (*r*=-0.031, *p*=0.893). Two eyes in this group experienced significant loss of vision (20/400); the cause for one eye was attributed to advanced glaucoma but was unexplained in the other eye. In contrast, in the gas group, no statistically significant change in CDVA was detected (*p*=0.786). Similarly, the gas group did not experience a statistically significant change in IOP, though in the SO group, the IOP increased by 0.7 mmHg (*p*=0.023), which is statistically significant (Fig. 5). By the time of the final follow-up, four eyes in gas group required topical anti-glaucoma treatment and none of them had advanced cupping. In contrast, topical IOP-lowering agents were being applied to seven of the eyes in the SO group. One of the eyes in this sub-group had advanced optic nerve cupping that had been treated by implanting an Ahmed glaucoma valve.

**Table 4:** Comparison of preoperative and postoperative visual acuity and intraocular pressure in the Silicone oil (SO) group and gas (SF6 or C3f8) group

**Parameter**			**Silicone Oil Group**	**Gas (SF6 or C3F8) Group**
CDVA (logMAR)				
	Preoperative	Mean ± SD	0.20±0.13	0.17±0.15
		Median (Range)	0.15 (0 to 0.40)	0.20 (0 to 0.60)
		Median Snellen (Range)	20/28 (20/20 to 20/50)	20/32 (20/20 to 20/80)
	Postoperative	Mean ± SD	0.33±0.35	0.16±0.10
		Median (Range)	0.30 (0 to 1.30)	0.20 (0 to 0.40)
		Median (Range)	20/50(20/20 to 20/400)	20/32 (20/20 to 20/50)
	*P*-value		0.051*	0.786*
IOP (mmHg)				
	Preoperative	Mean± SD	15.7±1.5	16.1±3.2
		Median (Range)	15.5 (13.0 to 18.0)	16.0 (10.0 to 25.0)
	Postoperative	Mean± SD	16.4±2.0	16.3±3.5
		Median (Range)	16.5 (13.0 to 20.0)	15.5 (12.0 to 28.0)
	*P*-value		0.023**	0.547*

*CDVA* corrected distance visual acuity, *LogMAR* logarithm of minimum angle of resolution, *SD* standard deviation, *SF*6 Sulfur hexafluoride, C3F8, Octafluoropropane

^*^Wilcoxon Signed Ranks Test

^**^Paired T-Test

## Discussion

This study compared the impact that gas and SO tamponades have upon the thickness of the retina and choroid, and visual acuity following PPV surgery for macula-on RRD. Prior to surgery, the grades of proliferative vitreoretinopathy in patients were similar in the two groups. The results indicate in the SO group eyes, macular retinal thickness declined during SO tamponade, with an apparent thinning of approximately 10-13% (29-42 µm) central macula, superior, inferior, nasal, and temporal when compared to preoperative retinal thickness of the same eye (Table 2). This finding aligns with data from other studies, demonstrating that heavy or regular use of SO tamponades often leads to retinal thinning compared to the untreated fellow eye [9, 13–16].

Our study design follows that of Christensen and la Cour [9]; but with a larger sample size (26 eyes of 26 patients in compared to 9 eyes of 9 patients underwent SO tamponade). Additionally, Christensen and la Cour’s utilized an earlier version of OCT, likely lacking automated thickness measurements, relying on additional software for thickness measurement as described in their study. Also, They did not measure retinal thickness outside the central 2 mm of the macula. However, our result demonstrates retinal thinning not only in the central macula but throughout the macula area in compared to their result where thinning was limited to the central 2 mm of the macula. Moreover, our study found that retinal thinning was generalized to inner and outer retina and not specific to the inner retina, as observed by Christensen et al. [9].

During SO tamponade, our results showed that 20% (5/24) of the eyes in the SO group exhibited a qualitative flattening of the foveal contour; however, this flattening resolved upon tamponade removal. This phenomenon, described by other researchers [6, 10], lacks a determined mechanism. It has been proposed that SO can initiate mechanical stretching of the foveal region, leading to premature loss of the outer nuclear layer [1, 17]. It is also possible that retinal thinning arises from emulsified SO entering the intraretinal space and harming retinal cells [18]. Additionally, low-molecular-weight components present in SO might diffuse into retinal tissue, acting as a toxin and stimulating inflammation which may induce thinning [19]. The removal of SO could potentially reverse some of these adverse effects, contributing to macular thickness restitution. Emulsification of SO, even subclinical, is believed to induce an inflammatory response and retinal degeneration [19]. Chen et al. recently demonstrated that such components could induce apoptosis of retinal cell lines [20]. The two eyes with severe visual loss during SO tamponade contributed to the overall effect of apparent retinal thinning in SO group, as both eyes experienced a significant reduction in macular retinal thickness.

Conversely, following PPV, retinal thickness in gas group in this study showed minimal thickening which wasn’t associated with cystoid macular edema, epiretinal membranes, or re-detachment. This observation aligns with previous reports [21]. A possible mechanism for this involves increased blood flow in the first few months post-PPV due to post-operative inflammation, particularly relevant when cataract surgery is performed alongside PPV, elevating the inflammatory cascade [21]. It is recognized that macular perfusion remains elevated for up to three months following phacoemulsification surgery [22, 23].

Regarding choroidal thickness following PPV, we observed minimal thinning of subfoveal choroidal thickness during SO tamponade, consistent with other studies [24, 25]. This thinning can be attributed to inflammatory response and transient low IOP during the surgery [24].

Our findings support other studies describing SO as producing poorer CDVA compared to gas tamponades [1, 9, 26]. Although the initial visual acuity of all eyes in the SO and gas groups was good, with at least 0.5 decimal, healthy macula with an intact ellipsoid zone and all eyes were completely reattached in a single surgery, post-operative CDVA in the SO group was reduced. Factors favoring SO over gas tamponade in our patients included the inferior location of the retinal tear, multiple retinal tears, and the need for air travel. However, recent trends suggest that long-acting gas may be preferable to regular SO in RRD with inferior breaks [27].

Although the average duration of SO tamponade was 8.1 months (±3.4 months), post-operative IOP was higher in the SO group, consistent with findings elsewhere [20].

The findings of this study are tempered by several limitations. As stated earlier, this was a retrospective study of eyes, thus there was no randomisation. Furthermore, decisions about which tamponade compound to use were made by surgeons at the time of surgery. The study did not address the variability of the duration of SO tamponade for outcome parameters other than CDVA, and the influence of SO emulsification was not considered. Also, we were not able to discriminate any potential influence of perfluoro-N-octane (PFO) on retinal or choroidal thickness.

On the positive side, the Spectralis OCT segmentation software's automated segmentation facility provided superb repeatability and reproducibility, generating accurate and clinically meaningful results without the need for manual modifications in measuring retinal thickness. The relationship between axial length (AL) and central retinal thickness has been studied previously [28, 29]. These studies did not find any correlation between the macular thickness and AL in myopic eyes, but only in highly myopic eyes with an AL greater than 25.5 or 26 mm [28]. However, highly myopic eyes with AL more than 26.1 mm were excluded from this study, thus this potential bias should not be relevant to this study. On the other hand, the potential hyperopic shift induced in phakic patients with SO tamponade raises questions about OCT image distortion and macular thickness measurement accuracy.

Further prospective randomized studies with larger patient cohorts and longer follow-ups are warranted. The incorporation of OCT angiography or fluoresceine angiography could establish whether the retinal thinning observed in this study is directly caused by SO or is an indirect result of changes to the retinal vasculature. Additionally, analyzing the retinal nerve fiber layer thickness could assess whether induced thinning during SO tamponade is contributed by high IOP, providing deeper insights into the mechanisms by which SO might be toxic to the human retina.

## Conclusion

This study used caliber measurement and automated segmentation facility of spectral domain OCT to confirm a reduction in the retinal and subfoveal choroidal thickness of eyes that had undergone PPV to repair macula-on RRD using SO tamponade in matching with gas tamponade. This thinning partially restored once the SO tamponade had been removed.

## Data Availability

The datasets used and/or analyzed during the current study available from the corresponding author on reasonable request.
